# The Onset Time of the Ownership Sensation in the Moving Rubber Hand Illusion

**DOI:** 10.3389/fpsyg.2017.00344

**Published:** 2017-03-10

**Authors:** Andreas Kalckert, H. H. Ehrsson

**Affiliations:** ^1^Psychology, University of Reading MalaysiaIskandar Puteri, Malaysia; ^2^Department of Neuroscience, Karolinska InstitutetStockholm, Sweden

**Keywords:** rubber hand illusion, sense of ownership, body perception, onset

## Abstract

The rubber hand illusion (RHI) is a perceptual illusion whereby a model hand is perceived as part of one’s own body. This illusion has been extensively studied, but little is known about the temporal evolution of this perceptual phenomenon, i.e., how long it takes until participants start to experience ownership over the model hand. In the present study, we investigated a version of the rubber hand experiment based on finger movements and measured the average onset time in active and passive movement conditions. This comparison enabled us to further explore the possible role of intentions and motor control processes that are only present in the active movement condition. The results from a large group of healthy participants (*n* = 117) showed that the illusion of ownership took approximately 23 s to emerge (active: 22.8; passive: 23.2). The 90th percentile occurs in both conditions within approximately 50 s (active: 50; passive: 50.6); therefore, most participants experience the illusion within the first minute. We found indirect evidence of a facilitatory effect of active movements compared to passive movements, and we discuss these results in the context of our current understanding of the processes underlying the moving RHI.

## Introduction

The rubber hand illusion (RHI) is a perceptual illusion whereby a fake model hand is perceived as part of one’s own body. This illusion arises when synchronous touches are applied to a rubber hand, in full view of the participant, and to the participant’s real hand, which is hidden from view (Botvinick and Cohen, 1998). After some time, most participants start to feel as if the touch originates from the model hand (referral of touch) and that the rubber hand is part of their own body (ownership). The RHI is a classical multisensory illusion that results from the dynamic integration of visual, tactile and proprioceptive information from the hand (Botvinick and Cohen, 1998; Samad et al., 2015). The illusion is typically measured by questionnaires wherein participants have to rate the feelings of ownership and referral of touch using visual analog rating scales as well as by more objective methods, such as registering the pointing error toward the model hand when participants are asked to manually indicate the location of their hands (“proprioceptive drift”) (Botvinick and Cohen, 1998) or skin conductance responses (Armel and Ramachandran, 2003) evoked by physical threats toward the rubber hand (Ehrsson, 2012). These methods try to evaluate the presence of the illusion by showing that the illusion is present in one illusion condition without doing so in the other control conditions. The illusion critically depends on the spatial and temporal congruency of the visual and tactile stimuli (Makin et al., 2008; Tsakiris, 2010; Ehrsson, 2012); therefore, contrasting synchronous visuotactile stimulation and asynchronous stimulation is one of the most commonly used comparisons to test the illusion in otherwise equivalent conditions.

**FIGURE 1:**
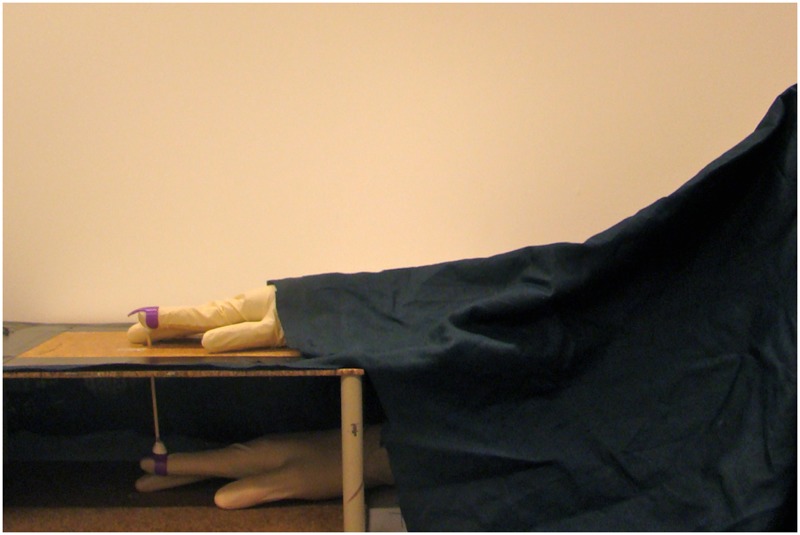
**Illustration of the moving rubber hand illusion setup.** Participants make brisk extension movements with their real index fingers (inside the box), which results in the corresponding movements of the rubber hand’s index finger (on top of the box). Both hands are covered with latex gloves.

Studies have also been conducted to investigate whether the illusion can be elicited with movements instead of with passive visuotactile stimulation (Tsakiris et al., 2006; Dummer et al., 2009; Kalckert and Ehrsson, 2012; Braun et al., 2014; Caspar et al., 2015; Jenkinson and Preston, 2015). In the current version of the moving RHI, the finger of a wooden model hand moves synchronously with the participant’s finger, which is hidden from view (**Figure 1**; Kalckert and Ehrsson, 2012). As in the classical RHI, participants develop a feeling of ownership over the hand when the seen and felt finger movements are synchronous, which does not occur when they are asynchronous (Kalckert and Ehrsson, 2014a). The moving version of the illusion depends on the integration of visual and kinesthetic information on the finger movements; however, unlike the classical version, it does not involve tactile stimulation from an external object touching the hand. The moving RHI can be induced with both active and passive finger movements. However, in the former case, motor control processes are additionally engaged, and the participants experience a sense of agency of the model hand’s movements, i.e., they feel that they are generating and controlling the movements of the rubber hand (Kalckert and Ehrsson, 2012).

At present, there is no reliable information on the temporal evolution of the moving RHI. However, this information would be relevant for both basic and applied research. From the perspective of cognitive science, a more detailed description of the temporal development of the illusion would enable researchers to estimate the minimal time needed to induce the illusion when designing their paradigms. Additionally, the onset time alone could also be used as a behavioral measure of the illusion. Moreover, the temporal evolution profile could provide information about the different processes involved in the illusion phenomenon. The elicitation of the illusion is thought to be preceded by multisensory recalibration processes during which visual and proprioceptive maps are realigned (Ehrsson et al., 2004), and this process could presumably be studied by using the onset-times as a dependent variable. From an applied perspective, new knowledge about the illusion onset time could be important for scientists who want to apply the principles of body ownership to the fields of virtual reality, teleoperation, and advanced prosthetic research. An important goal here is to learn to induce ownership onto various robotic and simulated body parts (Rosén et al., 2009; Slater et al., 2009; Marasco et al., 2011; Caspar et al., 2014; Ma and Hommel, 2015; Romano et al., 2015; Pavone et al., 2016).

With respect to the classical version of the RHI, we only have limited information about the onset of the ownership illusion. Ehrsson et al. (2004) reported that it took an average of 11.3 s (SD: ± 7.0 s) for participants to report a sensation of ownership (Ehrsson et al., 2004). Lloyd found that it took an average of 6.52 s for participants to report a referral of the touch sensation [see Figure 3B in Lloyd (2007)]. Therefore, we still have limited information about the time-course of limb-ownership illusions, and, to the best of our knowledge, no study has characterized the onset time of the moving RHI. Interestingly, anecdotal observations in our laboratory suggest that the moving RHI can sometimes arise after only a few seconds, suggesting that this manipulation of the experience of one’s own body can rapidly occur.

In the present study, we measured the onset time of the ownership illusion in the moving RHI in a large group of participants. We registered the time needed for repeated finger movements before the participants started to experience ownership over the model hand. We also compared active and passive movement conditions to learn more about the possible role of agency and motor control processes in the facilitation of the illusion.

## Materials and Methods

We tested 117 naïve participants (66 females, mean age = 24.3 years, SD ± 5.2, range 18–48). All subjects provided written informed consent in accordance with the Declaration of Helsinki. The protocol was approved by the Regional Ethical Review Board of Stockholm.

The behavioral paradigm used to induce the moving RHI followed our previous published protocols (Kalckert and Ehrsson, 2012; **Figure 1**). The participant sat at a table and put their right hand into a wooden box placed 30 cm in front of them. A life-sized wooden model of a human hand was placed on top of the box and was covered with a latex glove. The participant wore an identical latex glove on their right hand. The right hand of the participant was placed inside the box, 12 cm directly below the model hand. A cloth was placed over the participant’s right shoulder to cover the space between the model hand and participant. We first tested the participant using the moving RHI in the following four conditions, in line with our previous study (Kalckert and Ehrsson, 2012): active vs. passive movements and synchronous vs. asynchronous feedback. The participant’s right index finger was mechanically connected to the index finger of the model hand with a thin wooden stick and two rings attached to the fingertips. In the active synchronous condition, when the participant lifted their index finger, the model finger made the same movement in perfect synchrony. In the passive conditions, the participant relaxed their right hand and the experimenter generated the movements of the two fingers by pulling the stick upward (out of view of the participant). In the asynchronous conditions, the participant’s index finger and model finger were mechanically decoupled by “unlocking” the stick into two separate parts, and the experimenter then controlled all movements of the model hand’s index finger. Each trial lasted 2 min, and the participant either made active index finger taps at approximately 1 Hz speed or experienced the passive index finger taps at the same rate. In the asynchronous condition, the model hand’s finger movement was delayed approximately 500 ms with respect to the movements of the participant’s real index finger. Between each trial, participants had a 30–45-s break in which they removed the arm from the box and freely moved the arm and hand to relax and eliminate putative carry-over effects.

Each condition was tested once in one trial. The order of the trials was randomized across participants. After each condition, participants were asked to complete a short questionnaire that included statements of ownership [(1) “I felt as if I was looking at my hand” and (2) “I felt as if the rubber hand was my hand”] and control questions [(1) “It seems as if I had more than one right hand” and (2) “It felt as if I had no longer a right hand, as if my right hand had disappeared”]. The statements were rated on a Likert-scale from -3 to +3, where -3 means “strongly disagree,” +3 “strongly agree,” and zero uncertainty (“Neither agree or disagree”).

Participants who affirmed experiencing ownership of the model hand in the synchronous condition (Ownership rating ≥ 1) but not in the asynchronous (Ownership rating ≤-1) condition were tested again with the synchronous condition and asked to verbally indicate the time point at which they felt that “the rubber hand was my hand.” The experimenter timed this response with a stopwatch. We repeated the onset measurement three times to obtain an average onset time for each participant. Between each trial, participants had a 30–45 s break during which they were instructed to move their right hands to eliminate any remaining illusion before the next trial was started.

A subsample of participants also completed a questionnaire to measure delusional ideation by using the Peter’s Delusional Inventory (PDI, see Peters et al., 2004). These results have been published elsewhere (Louzolo et al., 2015).

## Results

Data were tested for normality using the Shapiro–Wilk test, and the appropriate parametric or non-parametric tests were used. All reported results are two-tailed unless otherwise stated.

**FIGURE 2:**
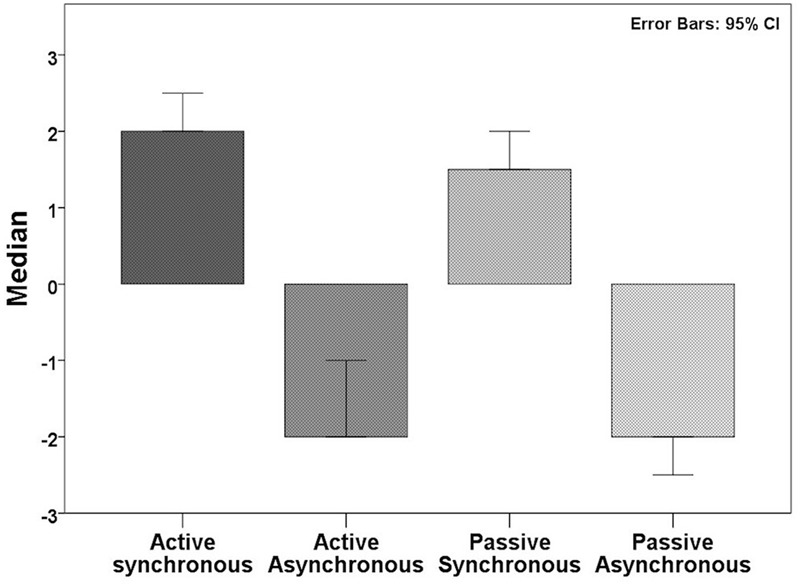
**Median ownership scores in the different conditions.** Participants experienced the illusion in the synchronous, but not asynchronous, condition.

First, we evaluated whether active or passive movements induced the illusion of ownership in our group of participants. For active synchronous movements, the ownership rating was positive (Median: 2.0) and significantly higher during synchronous compared to asynchronous movements (Median: -2.0; Wilcoxon: *Z* = -8.974, *p* < 0.000). We noted that in the active synchronous condition, approximately 80% of the participants (=94) affirmed ownership, i.e., gave a rating score of +1 or higher on the ownership related statement. Similarly, for passive synchronous movements, the ownership rating was positive (Median: 1.5) and significantly higher during synchronous compared to asynchronous movements (Median: -2.0; Wilcoxon: *Z* = -8.485, *p* < 0.000) (**Figure 2**). Descriptively, we observed that 76% (=89) of the participants affirmed ownership in the passive asynchronous condition (ownership score ≥ +1). Therefore, participants experienced a sense of ownership during synchronous, but not asynchronous, movements. The active synchronous condition was significantly higher than the passive synchronous condition (*Z* = -2.520, *p* < 0.012), although the effect size was small (*r* = 0.15).

**FIGURE 3:**
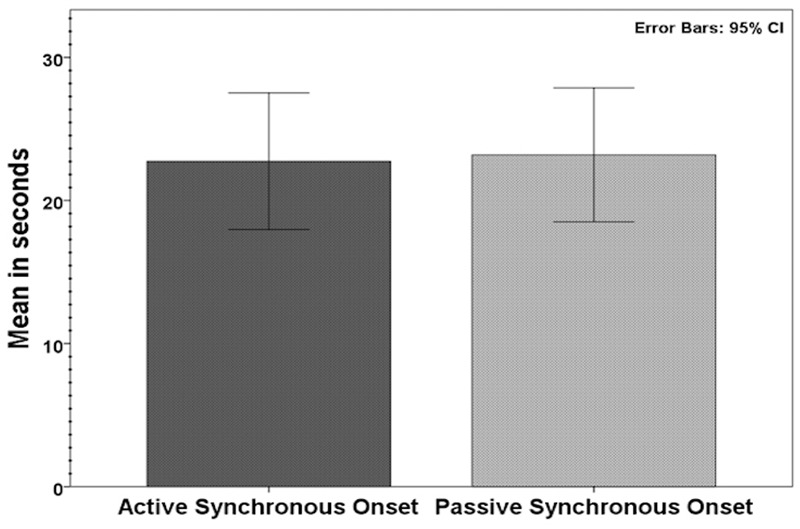
**Average onset times in the active synchronous (*n* = 60) and passive synchronous (*n* = 62) conditions**.

**FIGURE 4:**
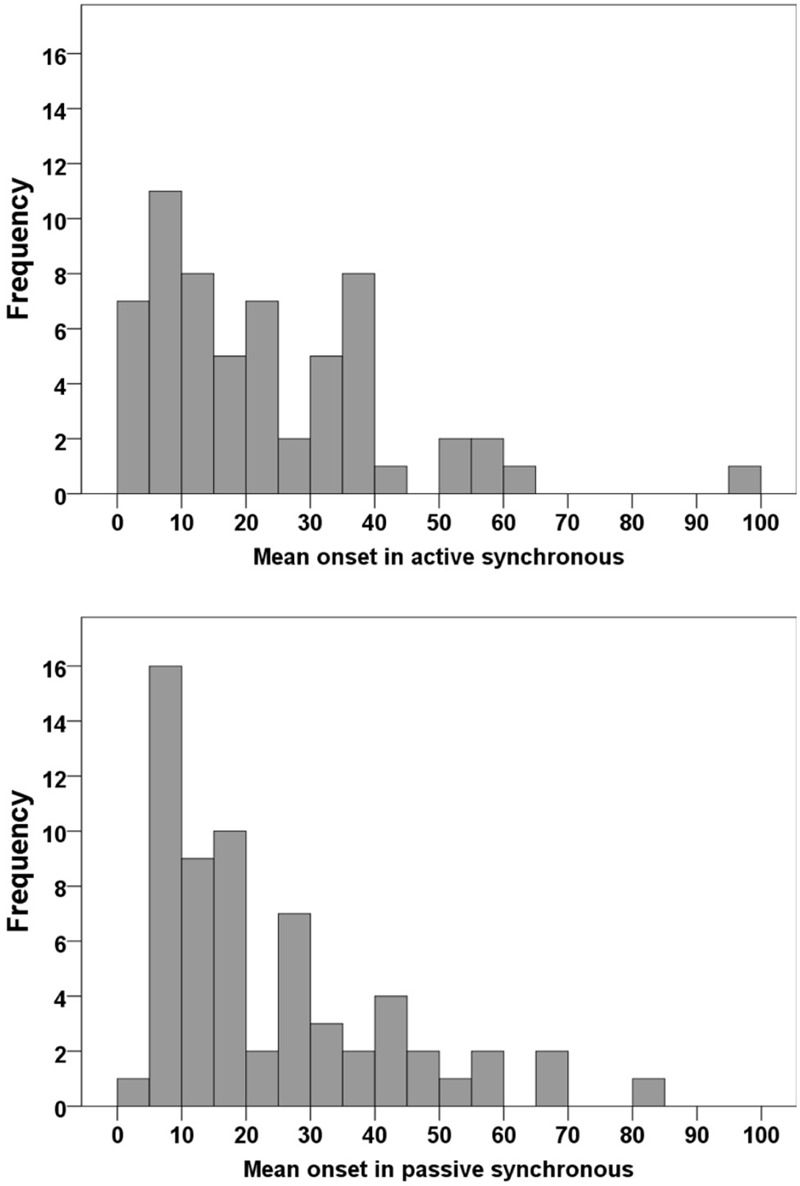
**Frequency distribution of the onset times in the active synchronous (top) and passive synchronous (bottom) conditions**.

Sixty participants who had affirmed the illusion according to our inclusion criteria (synchronous ≥ +1, asynchronous ≤-1; see above) were tested for the onset time in active synchronous movements. The average onset was 22.8 s (SD 18.5; range: 3.0–95.7). For passive movements, tested in 62 participants according to the same inclusion criteria, the average onset time was 23.2 s (SD 18.4; range: 3.7–82); see **Figure 3**. When comparing the average onset time in the two conditions, we found no significant difference (*Z* = -0.52, *p* = 0.958). Most participants experienced the illusion within the first minute; 97% in the active condition and 95% in the passive condition took less than 60 s to report the experience of ownership (**Figure 4**). Approximately 30% (active: 30%; passive: 27%) of the participants required less than 10 s.

We further repeated the analysis with those participants for whom we obtained an onset measurement in both active and passive conditions, allowing for a more sensitive within-participant comparison. In this subgroup of participants (*n* = 41), the active synchronous condition (Median: 2.5) was significantly rated higher in terms of ownership in the questionnaire than the asynchronous condition (Median: -2.0; *Z* = -5.598, *p* < 0.000). Similarly, the passive synchronous condition (Median: 2.0) was rated higher for ownership than the asynchronous condition (Median: -3.0; Z = -5.597, *p* < 0.000). The active synchronous condition was significantly rated higher than the passive synchronous condition (*Z* = -2.106, *p* = 0.035).

**FIGURE 5:**
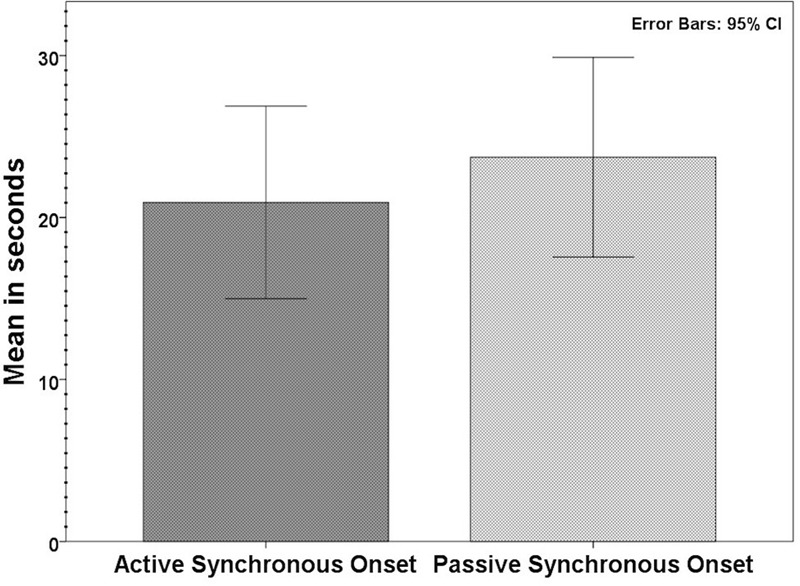
**Average onset times of the illusion in the subsample (*n* = 41) that experienced the illusion in both the active and passive synchronous conditions.** The difference in the onset times was significant (*p* < 0.05, see Results for details).

In terms of the onset of the illusion, the average onset time was 20.9 s (SD 18.8) for the active synchronous condition and 23.7 s (SD 19.5) for the passive synchronous condition. This difference was significant (*Z* = -2.469, *p* = 0.014), with a moderate effect size (*r* = 0.27); see **Figure 5**. We further observed that for 27 of these 41 participants, the onset time in the active synchronous condition was faster than in the passive synchronous condition.

**FIGURE 6:**
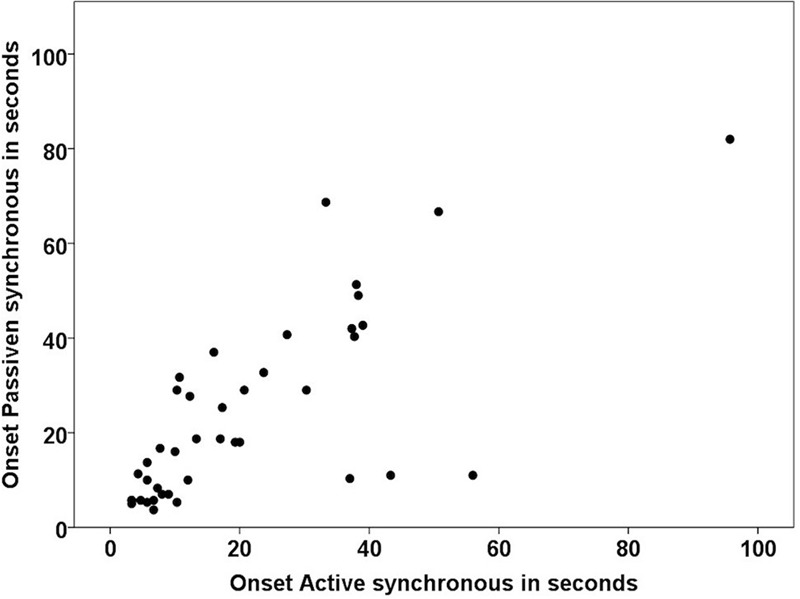
**Correlation between the onset times in the active and passive synchronous conditions in the subsample of participants affirming the illusion in both conditions (*n* = 41)**.

Additionally, we ran a correlation analysis between the onset times of active and passive synchronous movements and found that both were highly correlated (Spearman’s ρ: *n* = 41, *r* = 0.756, *p* < 0.000); see **Figure 6**.

## Discussion

In the present study, we tested for the onset times of the ownership sensation in the moving RHI. The active and passive finger movements took an average of 22.8 and 23.2 s, respectively. Most the participants, more than 95% in both cases, indicated that they felt the illusion within the first 60 s, and it was not uncommon to observe participants who felt the illusion in less than 10 s. When testing the subgroup of the participants who experienced the illusion equally in both the active and passive movement conditions, we found that in the passive condition, the illusion took significantly longer to elicit (23.7 vs. 20.0 s). These results support the notion of the relatively rapid onset of the moving RHI.

There is relatively little information available on the temporal evolvement of the RHI in the literature. To the best of our knowledge, only two studies explicitly reported the onset times for the classical version of the illusion. Lloyd (2007) observed that participants took an average of 6.52 s to experience a referral of touch sensation, whereas Ehrsson et al. (2004) found that it took 11.3 s for their group of participants to experience a sensation of ownership over the hand. In other variations of this experiment, such as the “somatic” version of the RHI in which blindfolded participants experience ownership of a rubber hand they touch, the illusion was triggered after 9.7 ± 5.3 s of stimulation (mean ± SD) (Ehrsson et al., 2005). Additionally, in the “invisible hand illusion,” in which participants experience ownership over a portion of empty space, participants reported this sensation after an average of 9.3 ± 5.0 s (mean ± SD) (Guterstam et al., 2013). However, these illusions did not involve any finger movements or motor intentions. Therefore, the present data add valuable information about the temporal development of the RHI phenomenon, and is the first to report on the onset times for the moving RHI.

Detailed information regarding the onset times of the various versions of the RHI would be valuable to applied researchers (**Table 1**). For example, there is substantial interest in the fields of virtual reality to use the principles found in bodily illusion research to create illusory ownership of simulated bodies and limbs in virtual reality applications (Slater et al., 2009; Ma and Hommel, 2013; Maselli and Slater, 2013; Padrao et al., 2015; Pavone et al., 2016). In these applications, it is desirable to induce the illusion as quickly as possible to enable the user to gain rapid experience of the virtual reality. To the best of our knowledge, only one study directly assessed the onset of the ownership sensation in a virtual hand illusion using visuo-tactile stimulation (Perez-Marcos et al., 2011). In that study, it took an average of at least 36 s for participants to report the ownership illusion (see Supplementary Table 3 in Perez-Marcos et al., 2011), which is slower than previous reports on the illusion onset times in the classical RHI (Ehrsson et al., 2004; Lloyd, 2007) as well as slower than the onset times reported here for the moving RHI. This result contrasts with anecdotal reports of ownership illusions of full body avatars, which apparently can be experienced almost instantaneously. However, it is difficult to compare the present results directly with studies of full-body avatars in virtual reality. Obviously, the use of head-mounted displays and computer-generated graphics is one difference. However, more importantly, in many virtual reality experiments, the location of the virtual hand is directly superimposed on the location of the real hand without the 12 cm distance between the hands, as in our experiments (Perez-Marcos et al., 2011; Kilteni et al., 2012; Padrao et al., 2015). Moreover, virtual reality experiments often use head tracking and tracking of proximal arm movements, so that when the subject moves his or her head or arm, the environment and simulated arm also move accordingly (Slater et al., 2009; Sanchez-Vives et al., 2010; Kilteni et al., 2012; Pavone et al., 2016; Bourdin et al., 2017). Therefore, in virtual reality experiments, there typically are more multisensory cues that could facilitate ownership than in our setup. Nevertheless, our results suggest that when there is a spatial discrepancy between the locations of the artificial hand in view and the unseen real hand, the illusion takes some time to build up. In cases when discrepancies are too large, on the order of 30 cm or more, the illusion is abolished (Kalckert and Ehrsson, 2014b). We suggest that future studies should investigate whether the onset times can be used as an additional parameter to further characterize the illusion of ownership in virtual reality applications, especially those whereby the observed location of the virtual hand is not the same as the location of the real one.

**Table 1:** Percentiles for the onset times in the two synchronous conditions.

Percentiles	Active synchronous	Passive synchronous
25	7.4	7.8
50	**19.3**	**18.3**
75	35.1	32.8
90	50.0	50.6

*The median onset time for active synchronous movements is 19.3 s; for passive synchronous movements, it is 18.3 s.*

Similarly, this knowledge can also be relevant for the design of advanced prosthetic devices for upper limb amputees (Ehrsson et al., 2008; Rosén et al., 2009; Marasco et al., 2011), particularly for those based on active movement control (Velliste et al., 2008). Studies have shown that the multisensory principles of the RHI can be successfully applied to robotic hand-like devices, which can be controlled by the user. The experience of these robotic devices can be associated with sensations of ownership and agency (Caspar et al., 2014; Braun et al., 2016; Romano et al., 2015), particularly once these include different forms of somatosensory feedback by peripheral or central stimulation (Raspopovic et al., 2014; Collins et al., 2017). Researchers need to consider that the participant needs a certain time of stimulation before they can assume successful induction of the illusion.

The present results can help to determine the minimum time needed to induce the illusion in most participants. This information is relevant for both applied research and studies investigating the illusion in general. In previous studies, the duration in which repeated multisensory stimulation is delivered to induce the RHI can vary widely in different experiments, from approximately 40 s (Ehrsson et al., 2004) to up 10 min in some cases (Botvinick and Cohen, 1998). In our study, most participants (active: 95%; passive: 97%) reported the sensation of ownership within 60 s (**Figure 4**). Therefore, future experiments can design experiments with a duration of 60 s to reliably induce the moving RHI in most participants, allowing for more efficient experimental designs.

However, it is not clear why participants differ in their responsiveness to the illusion, i.e., why some participants experience the illusion within the first few seconds, whereas other participants need more than 60 s before the illusion starts and some never experience it. Previous studies of the classical rubber hand paradigm found that between 60 and 80% of participants experience the illusion depending on the criteria used to conclude successful induction of the illusion (Lloyd, 2007; Slater et al., 2008; Petkova and Ehrsson, 2009). This typical percentage of illusion responders is very similar to our previous observations with the moving RHI (Kalckert and Ehrsson, 2014a). It is not clear what determines the inter-individual differences in the RHI, although one hypothesis is that it might reflect individual differences in the relative weighting of different sensory modalities in the integration process (Kilteni et al., 2015; Samad et al., 2015). A higher weighting on vision should facilitate the illusion, whereas a higher weighting on proprioception should work against the illusion. Onset times may very well provide a complementary measure to determine the differences between individuals in the RHI paradigm that might be more sensitive than the typically used seven point Likert-scale.

The present results reinforce our earlier conclusions that the RHI can be elicited with both active and passive movements and that the illusion is probably the same, or at least very similar (but see the next paragraph), in these two cases. Both the active and passive illusions were well affirmed in the questionnaire data (80% or 76%, respectively, have an ownership score of ≥ 1 in the synchronous conditions) and, eliminated by the asynchronous condition, had similar onset-times in the larger group of participants (*n* = 60), and the onset-times were significantly correlated. This result suggests that a genuine ownership illusion is triggered in both conditions and by the same type of visuo-proprioceptive integration process. This result is also consistent with previous experiments that directly compared the RHI with active or passive movements to the classical version with the application of brushstrokes (Kalckert and Ehrsson, 2014a). That is, consistent with the idea that different types of multisensory correlations can elicit the illusion (Ehrsson et al., 2005; Tsakiris et al., 2006; Walsh et al., 2011; Guterstam et al., 2013).

Nevertheless, whether the motor control processes influence the moving RHI is still an important unanswered question. The critical differences between active and passive movements are the involvement of (i) motor intentions, (ii) comparisons between the predicted sensory consequences of the movement based on efference copy and the actual sensory feedback, and (iii) the sense of agency. These processes, which are intimately linked to motor control, are only present in the active condition of the moving RHI. Therefore, the fundamental question here is whether these motor-related processes contribute to the emergence of the illusion by influencing the multisensory integration of visual, tactile and proprioceptive information. By comparing active and passive movements, we can directly contrast conditions that are matched in terms of visual and proprioceptive feedback, while differing in terms of agency and motor control processes (Kalckert and Ehrsson 2012; 2014a). Recent studies, including our own, show a mixed picture in terms of the results from comparing the strength of the illusion in active and passive conditions. Some studies reported a stronger illusion experience (questionnaire ratings) in active movements than in passive movements (Braun et al., 2014; Jenkinson and Preston, 2015), and some found a stronger illusion experience in the passive movement condition (Walsh et al., 2011). Other studies found no difference when they quantified the illusion with the proprioceptive drift (Tsakiris et al., 2006). In our previous studies, we found mixed results. In Kalckert and Ehrsson (2012), active movements produced significantly higher affirmative ownership ratings in the active condition than for the passive condition, but this difference was relatively small and not reproduced in the proprioceptive drift. In a later study, we found no significant difference in the ownership ratings between active synchronous movements, passive synchronous movements, and a classical condition with synchronous brush stroking (Kalckert and Ehrsson, 2014a). Moreover, a similar proprioceptive drift was observed in the classical condition and active movement condition (Kalckert and Ehrsson, 2014a).

Therefore, it is interesting that we, in the present study, found significantly higher ownership scores in the active condition compared to the passive condition. This significantly stronger illusion in the active condition is also reflected in the significantly faster onset times for active movements in the subsample of participants who experienced the illusion in both synchronous conditions. One possible explanation for this result could be that the integration of visual and somatosensory feedback changes depending on whether the feedback is matched by a sensorimotor prediction (active movements) or not (passive movements). Motor control processes can affect basic sensory processes through efference copy mechanisms (von Holst and Mittelstaedt, 1950; Bays and Wolpert, 2006). Most notably, somatosensory perception is attenuated during self-produced touches compared to externally produced touches via central cancelation mechanisms that are thought to involve sensory prediction mechanisms in the cerebellum (Blakemore et al., 1998; Shergill et al., 2003). Future experiments need to determine the possible contribution of agency and motor control processes in ownership illusions, and these experiments could benefit from including illusion onset timings as part of the dependent data.

Our results naturally draw upon some assumptions. We applied a strict criterion for which participants were subsequently tested in the onset measurement. We chose to include participants with an ownership score of ≥ 1 in the synchronous and ≤-1 in the asynchronous condition. The threshold values may be defined differently, but we introduced these for a practical reason, which is that the difference between synchronous and asynchronous stimulation is one of the most observed and reliable effects in RHI experiments (Botvinick and Cohen, 1998; Tsakiris et al., 2006; Kalckert and Ehrsson, 2012). We measured the illusion onset in participants who could clearly distinguish the two different situations of “owning” and “not owning” the model hand. Therefore, when a participant had a clear experience of the ownership illusion in the synchronous condition, we could refer to this ownership experience and explain to participants that we were looking for the appearance of this particular sensation in the onset timing experiments, which makes this approach more reliable because the illusion can be associated with a variety of different subjective experiences (Longo et al., 2008).

The present results add to the limited literature on the temporal evolution of the rubber had illusion. The temporal onset of the illusion can provide a new objective approach for reporting the illusion beyond the typical questionnaire, proprioceptive-drift and GSR based measurements that have mainly been used so far in the literature. Onset times should also be particularly valuable for future applied research, for which this information can be incorporated in the design of a variety of applications, such as advanced prosthetic limbs. Further research is needed to investigate potential differences between the different versions of the illusion and the role of the different processes, such as multisensory integration and motor control processes, in the experience of ownership of a moving body.

## Author Contributions

AK and HE designed the experiments. AK conducted the experiment. AK analyzed the data. AK and HE wrote the manuscript.

## Conflict of Interest Statement

The authors declare that the research was conducted in the absence of any commercial or financial relationships that could be construed as a potential conflict of interest. The reviewer PSJ and the handling Editor declared their shared affiliation, and the handling Editor states that the process nevertheless met the standards of a fair and objective review.
